# Daikenchuto improves methotrexate-induced chronic small intestinal mucositis by promoting angiogenesis

**DOI:** 10.3389/fphar.2025.1623726

**Published:** 2025-08-21

**Authors:** Peilin Li, Yusuke Inoue, Daichi Sadatomi, Sachiko Mogami, Daisuke Miyamoto, Toshiyuki Adachi, Tomohiko Adachi, Akihiko Soyama, Kengo Kanetaka, Weili Gu, Susumu Eguchi

**Affiliations:** ^1^ Department of Surgery, Nagasaki University Graduate School of Biomedical Sciences, Nagasaki, Japan; ^2^ Department of Surgery, Guangzhou First People’s Hospital, School of Medicine, South China University of Technology, Guangzhou, Guangdong, China; ^3^ TSUMURA Kampo Research Laboratories, Research and Development Division, TSUMURA & CO., Ibaraki, Japan

**Keywords:** chemotherapy, methotrexate, intestinal mucositis, daikenchuto, angiogenesis

## Abstract

**Aim:**

Chronic small-intestinal mucositis (CIM) is a severe gastrointestinal complication that has limited treatment options. This study investigated the potential therapeutic effects of Daikenchuto (DKT), a traditional medicine, on mitigating methotrexate (MTX)-induced CIM in rats.

**Methods:**

Male Sprague-Dawley rats were assigned to four groups: control, MTX, DKT-MTX, and DKT. CIM was induced by intraperitoneal administration of MTX (10 mg/kg every 6 days), while DKT (2.7% wt/wt) was orally administered via feed. The surviving rats were euthanized on day 60. Rat intestinal epithelial cells (IEC-6) were used to examine DKT’s cytoprotective effects *in vitro*.

**Results:**

DKT treatment improved survival, reduced gastrointestinal symptoms, and alleviated histological damage, including villus atrophy and crypt hyperplasia. DKT restored mucosal integrity by enhancing the expression of tight junction proteins (CLDN-3) and nutrient transporters (B0,+AT, EAAT3), and by reducing oxidative stress and epithelial cell death. Furthermore, DKT promoted mucosal angiogenesis, as evidenced by increased expression of CD34, VEGFR2, and VEGFA in both tissues and cells. qRT-PCR confirmed upregulation of genes associated with angiogenesis, barrier repair, and mucosal regeneration.

**Conclusion:**

DKT exerts protective effects against MTX-induced CIM by enhancing angiogenesis, promoting epithelial regeneration, and restoring mucosal barrier function. These findings suggest DKT as a promising adjunctive therapy for managing chronic intestinal toxicity induced by chemotherapy.

## 1 Introduction

The small and large intestines are among the most common sites of adverse drug reactions, accounting for 20%–40% of all drug side effects (Zeino et al., 2010). Chemotherapy-induced intestinal mucositis is a common, complex, and severe gastrointestinal complication that represents a prevalent off-target toxicity (Akbarali et al., 2022). In addition to antineoplastic agents, which include gastrointestinal drugs, non-steroidal anti-inflammatory drugs (NSAIDs), and antibacterial drugs, these drugs directly stimulate the gastrointestinal mucosa, inhibit the protective mechanism of the gastrointestinal tract, and change the gastrointestinal flora and microenvironment, thus causing gastrointestinal side effects (Watanabe et al., 2020; Hamdeh et al., 2021). These gastrointestinal side effects include mucositis, ulcers, and diarrhea, which lead to a decreased quality of life, treatment intolerance resulting in discontinuation, and even death (Hamdeh et al., 2021). Aside from severe acute gastrointestinal damage leading to drug cessation, a more prevalent issue arises from prolonged oral medication and multiple cycles of chemotherapy induce chronic small-intestinal mucositis (CIM). CIM diminishes the quality of life of patients and exacerbates the suffering experienced throughout the course of treatment. In this process, it is particularly important to use low-irritation gastrointestinal protective drugs to improve the gastrointestinal side effects of patients. However, relevant drugs have not received much attention.

Methotrexate (MTX), an inhibitor of dihydrofolate reductase and DNA synthesis, is widely used in cancer chemotherapy and in the treatment of rheumatoid arthritis. It is an immunosuppressant that causes morphological damage to the small intestinal mucosa and induces severe intestinal barrier dysfunction, which leads to acute and chronic small intestinal mucositis (Carneiro-Filho et al., 2004). In a randomized clinical trial, long-term low-dose (median weekly dose 16 mg) MTX as the first-line drug for the treatment of rheumatoid arthritis was associated with small to moderate elevation in risk of skin cancer and gastrointestinal, infectious, pulmonary, and hematologic adverse effects (Solomon et al., 2020). A dose–toxicity relation was seen in the odds ratios for patients with adverse events, gastrointestinal and mucocutaneous toxicity, with the highest odds ratios for 25–35 mg/week methotrexate (Visser and van der Heijde, 2009). The treatment dose of MTX in rheumatoid arthritis is typically a long-term low dose, while in oncology, higher doses are required, but this is associated with more severe complications, particularly gastrointestinal adverse events (O'Donoghue et al., 2022). Damage to the mucosa is dose-dependent, and the use of larger or incorrect doses of MTX in cancer chemotherapy causes severe mucositis that can even lead to death (Maagdenberg et al., 2021). In general, animal models of severe and acute small intestinal mucositis usually use small doses of MTX for intraperitoneal injection once every 3 days (Solomon et al., 2020; Li et al., 2023a). When used in small weekly low doses for the treatment of rheumatoid arthritis, it may be related to chronic damage to the small intestinal mucosa (Solomon et al., 2020; Ozcicek et al., 2020). Acute and chronic injuries of the small intestine induced by MTX have received great attention and research, but there is a lack of simple and effective means to prevent, protect, and treat CIM caused by MTX.

Daikenchuto (DKT) is a traditional medicine containing Japanese pepper (the fruit of *Zanthoxylum piperitum Benn*), processed ginger (the rhizome of *Zingiber officinale Rosc.*), ginseng (the radix of *Panax ginseng C.A. Mey*), and malt sugar powder (the malt sugar derived from *Hordeum vulgare* L.). It improves gastrointestinal motility, has anti-inflammatory properties, increases intestinal blood flow, and impacts the intestinal microbiome (Suzuki et al., 2021; Wada et al., 2022). DKT enhances gastrointestinal motility by modulating intestinal contraction and relaxation through the release of acetylcholine, nitric oxide, and excitatory neurotransmitters (Kogure et al., 2020). Its anti-inflammatory effects involve the downregulation of COX-2, upregulation of endogenous adrenomedullin (ADM), and suppression of eosinophil infiltration (Kono et al., 2013). DKT regulates intestinal blood flow by stimulating epithelial TRPA1, leading to the release of endogenous ADM (Kono et al., 2013). A study also demonstrated that DKT can suppress the side effect of severe diarrhea associated with irinotecan hydrochloride and that it can improve the barrier functions by increasing the expression of zonula occluden-1 (ZO-1), occludin and claudin-4 (CLDN-4) (Takasu et al., 2017). Recent studies have also shown that DKT can change the gastrointestinal flora in patients after gastrointestinal and hepatic surgery, thus providing long-term improvement in dysbiosis and gastrointestinal dysfunction (Kono et al., 2022). In addition, DKT has been widely used in the clinical setting in the treatment of patients with gastrointestinal symptoms, such as postoperative intestinal obstruction, inflammatory bowel disease, abdominal pain, and abdominal distension (Kono et al., 2022; Eguchi et al., 2021). Our previous study on the use of DKT in MTX-induced acute small intestinal mucosal injury showed that DKT could reduce MTX-induced small intestinal mucosal injury and improve the mucosal barrier function (Li et al., 2023b).

Current CIM also lacks specific medications, which are typically managed by dose reduction or discontinuation. Considering DKT’s potential mechanisms of action in recent studies and our clinical experience, we hypothesize that DKT may ameliorate MTX-induced CIM. This study aimed to explore the potential therapeutic effect and mechanism of action of DKT in improving MTX-induced CIM and facilitating recovery.

## 2 Methods

### 2.1 Animals

Male Sprague-Dawley rats (age: 7 weeks, 160–190 g; CLEA Japan Inc., Tokyo, Japan) were used in this study. The rats were bred and kept in conventional rat enclosures following a 12-h cycle of light and darkness, with unrestricted access to both water and rat feed. All animal experiments adhered to protocols approved by the Institutional Animal Care Committee of Nagasaki University and were conducted in accordance with the pertinent guidelines and regulations of Nagasaki University. The study was conducted in accordance with the ARRIVE guidelines.

### 2.2 Preparation and pharmacokinetics of DKT

DKT (Lot ID:2180100010, 2220100010) was manufactured by TSUMURA and CO. (Tokyo, Japan) as the intermediate product (extract powder) of ‘TSUMURA Daikenchuto Extract Granules for Ethical Use’ as shown in their website (https://www.tsumura.co.jp/english/kampo/, and https://www.tsumura.co.jp/english/ir/library/integrated-report/). The extract quality was standardized based on good manufacturing practices, as defined by the Ministry of Health, Labour, and Welfare of Japan and are in accordance with the Japanese Pharmacopoeia (JP) (https://www.pmda.go.jp/english/rs-sb-std/standards-development/jp/0029.html, https://www.mhlw.go.jp/content/11120000/000904450.pdf). Briefly, 15 g of DKT formulations consist of a mixture of a medicinal botanical extract powder (1.25 g) and maltose (10 g) (Munekage et al., 2011). The formulation used in this study was manufactured by spray-drying the hot-water extract of the following three crude drugs, in accordance with the Japanese Pharmacopoeia (JP): five parts of JP *Z. officinale* Roscoe [Zingiberaceae; *Zingiberis rhizoma processum*], 2 parts of JP *Z. piperitum* (L.) DC. [Rutaceae; *Zanthoxyli pericarpium*], and 3 parts of JP *P. ginseng* C.A. Meyer [Araliaceae; *Ginseng radix*] (Kono et al., 2015). The previous study was also conducted with the drug sample for Tsumura, which they have the standard production with GMP. The major ingredients in DKT contained hydroxy α-sanshool, hydroxy β-sanshool, α-sanshool, β-sanshool, γ-sanshool, 6-gingerol, 6-shogaol, 10-shogaol and 6-gingesulfonic acid, which were determined by three-dimensional high performance liquid chromatography (HPLC) as previously reported (Tokita et al., 2007). Also, the plasma concentrations of bioactive metabolites including hydroxy α-sanshool, hydroxy β-sanshool, 6-shogaol, and 10-shogaol reached the maximum concentration (approximately 400, 80, 0.14, and 0.6 ng/mL, respectively, after a 5-g administration of DKT within 30 min after administration, and the mean half-life was approximately 2 h (Munekage et al., 2011).

### 2.3 Experimental protocol

All animals were randomly assigned to one of four groups: control (Con, n = 6), MTX-induced model (MTX, n = 6), DKT treatment (DKT-MTX, n = 6), and DKT-only (DKT, n = 6). Intraperitoneal injections of MTX (10 mg/kg every 6 days) were administered to the MTX and DKT-MTX groups. The DKT dose used in this study was based on the clinically approved human dosage. In clinical practice, adults are typically prescribed 15 g of DKT per day. To simulate this exposure in rats, 27g DKT powder/Kg (2.7 % wt/wt) was incorporated into the standard rodent diet, as previously described (Hirose et al., 2018; Kubota et al., 2019). This oral administration route was chosen to reflect the clinical mode of DKT delivery and ensure continuous systemic exposure throughout the study period. For the DKT-MTX and DKT groups, 2.7% of the total mass of DKT (from Tsumura and Co., Tokyo, Japan) was incorporated into the feed (CE-2 feed; CLEA Japan Inc.) and administered orally from the initiation of MTX administration. The control group received intraperitoneal injections of an equivalent volume of normal saline at equal intervals.

The weight of each rat was documented every 6 days during the experiment. After obtaining small intestinal tissues using deep isoflurane respiratory anesthesia (Wako Pure Chemical, Osaka, Japan) with an All-in-one Anesthetizer (Muromachi Kikai Co. LTD, Japan), all surviving rats were humanely euthanized on day 60 through severance of the vena cava, leading to exsanguination.

### 2.4 Intestinal histology

The rats were sacrificed under anesthesia on day 60, and small intestinal tissues were collected immediately. The rat small intestinal tissue was fixed with 4% paraformaldehyde in phosphate-buffered solution (PBS; Wako Pure Chemical, Osaka, Japan) for 3 days. Fixed tissues were embedded in paraffin, cut into 5-μm sections, and deparaffinized for standard histological staining with hematoxylin and eosin (H&E). Hematoxylin-stained sections were evaluated blindly for intestinal inflammation, which included crypt length, architecture and abscesses, loss of goblet cells, tissue damage, and infiltration of leukocytes and neutrophils.

For immunohistochemical staining, tissue sections of the small intestine were stained for neutrophil myeloperoxidase (MPO), diamine oxidase (DAO), and CD34 (see S1 Table for antibody information). The staining procedures were performed according to the recommended protocols. Immunofluorescence staining of the small intestine tissue sections included antibodies against CD31, VEGFR-2, KI-67, ZO-1, CLDN-3, and VEGFA (Supplementary Table S1). Fluorescence and bright-field images were captured using a microscope (Ti-U and C-HGFI, Nikon, Tokyo, Japan). For each marker, at least 10 randomly selected high-power fields per sample were captured under identical exposure settings to ensure consistency. The percentage of the total area of the small intestinal sections was measured in at least 10 positions using the ImageJ software program (NIH, USA). All analyses were conducted in a blinded manner by two independent observers.

### 2.5 Quantitative real-time polymerase chain reaction (QRT-PCR)

Tissue samples were acquired at defined time points for mRNA extraction using spin columns, according to the manufacturer’s instructions (NucleoSpin RNA II; Macherey-Nagel, Duren, Germany). cDNA was synthesized from the total RNA using a high-capacity cDNA reverse transcription kit (Applied Biosystems, Tokyo, Japan). Briefly, qRT-PCR amplification was performed using Applied Biosystems (Taq-man is listed in Supplementary Table S2). The gene expression was normalized to that of GAPDH (control intestinal tissue was set at 1.0), and the mRNA expression was determined using the comparative cycle time (ΔΔCt) method.

### 2.6 Cell culture

Rat intestinal epithelial cell line IEC-6 cells (DS Pharmaceuticals, Osaka, Japan) were cultured in Dulbecco’s Modified Eagle Medium (DMEM) supplemented with 100 units/mL penicillin G, 0.1 mg/mL streptomycin, 5% fetal bovine serum at 37 °C and 5% CO_2_ atmosphere.

### 2.7 Cell treatment

IEC-6 cells were seeded in 96-well plates. The following day, cells were treated with MTX in combination with DKT for 24 h. The DKT concentrations used for *in vitro* experiments (1-10-100 μg/mL) were selected based on previously published dose–response studies and preliminary cytotoxicity screening in intestinal epithelial cells (Kono et al., 2010).

When evaluating barrier integrity, IEC-6 cells were seeded in 24-well Millicell^®^ Transwell inserts (Merck Millipore, MA, USA) and cultured for 1 week to form confluent monolayers. Cells were then treated with MTX alone or co-treated with DKT for 24 h.

### 2.8 Cytotoxicity assay

After the cell treatment, culture supernatants were collected, and cytotoxicity was assayed using the LDH-Cytotoxic Test Kit (Fujifilm Wako, Osaka, Japan). To obtain a positive control, cells were treated with 0.2% Tween 20 for 15 min, and the supernatants were collected. Cytotoxicity was calculated using the following formula for relative LDH activity: Cell death (%) = 100 × a/b, where a = absorbance at 560 nm of the test sample, b = positive control.

### 2.9 Reactive oxygen species measurement

After the treatment, cells were incubated at 37 °C in the dark with 5 μM CM-H_2_DCFDA (Thermo Fisher Scientific, MA, USA) for 30 min and fluorescence intensity was measured using a fluorescence microplate reader (INFINITE200 PRO, TECAN, Mannedorf, Switzerland, ex: 495 nm, em: 530 nm).

### 2.10 Cell proliferation activity assay

To evaluate cell proliferation activity, cells were loaded with 8 μL of Cell Counting Kit-8 reagents (CCK-8; Dojindo Laboratories, Kumamoto, Japan) and the plates were incubated at 37 °C. Cell proliferation activity is presented as the change in absorbance at 450 nm, as determined using a microplate reader (SpectraMax Plus 384, Molecular Devices, CA, USA).

### 2.11 Barrier integrity assessment

Barrier integrity was assessed by evaluating the permeability of FITC-dextran (MW 40,000; Sigma-Aldrich, MO, USA) across the cell monolayer. Following the treatment, FITC-dextran (1 mg/mL) containing medium was added to the apical chamber and cells were incubated at 37 °C for 1 h. After the incubation, basolateral medium was collected and transferred to a 96-well black plate for fluorescence measurement. The fluorescence intensity was measured using a fluorescence plate reader (INFINITE200 PRO, ex: 495 nm, em: 530 nm).

### 2.12 Amino acid uptake assay

After the treatment, cells were incubated with 50 μM β-Ala-Lys (AMCA) (Peptide Institute, Inc., Osaka, Japan) at 37 °C for 1 h. To remove residual extracellular regents, the cells were washed three times with PBS. Subsequently, the cells were lysed with RIPA buffer and analyzed using a fluorescence plate reader (INFINITE200 PRO, ex: 350 nm, em: 455 nm).

### 2.13 Statistical analyses

Each experimental group consisted of at least six rats. The qRT-PCR data represent the mean ± standard error of the mean from three biological replicates, with mRNA collected from at least three samples. Immunohistochemical positivity was determined by analyzing at least 10 positions using ImageJ (ImageJ 1.53k, NIH, USA). Data are expressed as the mean ± S.E.M. Statistical analyses were performed using GraphPad Prism (GraphPad Software, Inc., California, USA) with a one-way ANOVA, t-test, or repeated measures ANOVA, as appropriate. Survival rates were analyzed using the log-rank (Mantel-Cox) test, with significance denoted by asterisks (*P < 0.05, **P < 0.01) in t-tests or a one-way ANOVA.

## 3 Results

### 3.1 DKT reduced the gastric symptoms and increased the survival rate of rats with chronic small intestinal injury induced by MTX

**FIGURE 1:**
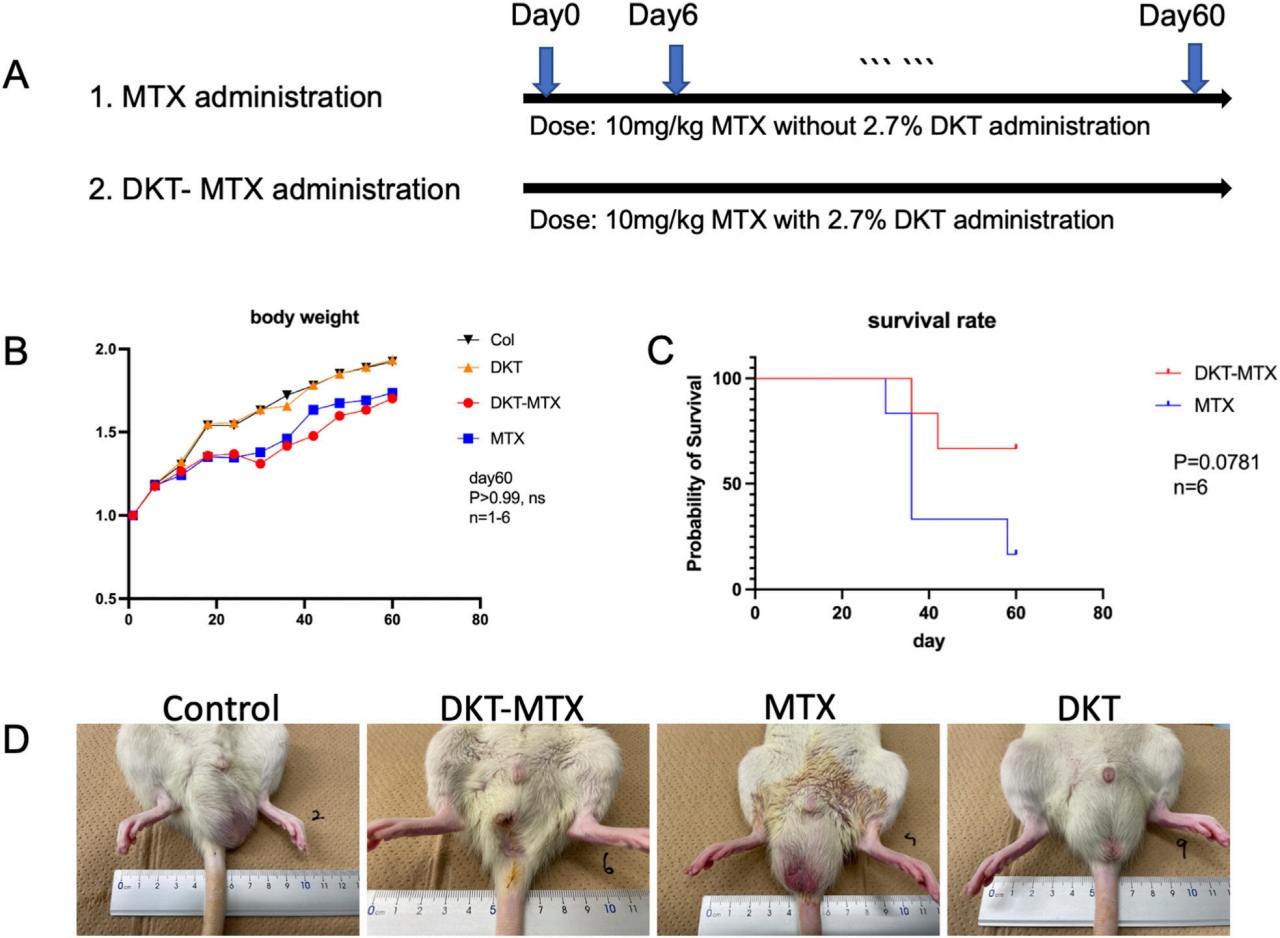
DKT reduced gastric symptoms and increased the survival rate in rats with chronic small intestinal injury induced by MTX. **(A)** The experimental procedure. **(B)** The body weight changed in comparison to the control group, MTX group, DKT-MTX group, and DKT group, there was no significant difference between the MTX and DKT-MTX groups (ns, day 60, n = 1-6). **(C)** There was a trend toward a difference in the survival rate of the MTX and DKT-MTX groups (P = 0.0781, n = 6, the Log-rank (Mantel-Cox) test). **(D)** MTX induced intestinal mucosal damage and diarrhea in rats.

MTX was administered to induce CIM, with an optimal dose and frequency of 10 mg/kg administered every 6 days (Figure 1A; Supplementary Figure S1). The experimental groups and subsequent administration of DKT were organized according to the flowchart in Figure 1A. In accordance with previous reports and the prescribed dosage regimen for patients, oral administration of DKT was initiated by incorporating 2.7% of the total mass into the feed (Li et al., 2023b). After 60 days of drug induction and concurrent DKT treatment, the body weight of rats in both the MTX and DKT-MTX groups exhibited a decreased trend, but no significance difference compared to the control and DKT groups in body weight between the MTX and DKT-MTX groups (Figure 1B). After the entire 60-day treatment period, the survival rate of rats in the MTX group was lower than that of rats in the DKT-MTX group (P = 0.0781) (Figure 1C). Regarding gastrointestinal symptoms, the presence and severity of diarrhea were assessed, with rats in the MTX group manifesting conspicuous watery diarrhea, while rats in the DKT-MTX group exhibited mild diarrhea during this period (Figure 1D; Supplementary Figure S2).

### 3.2 DKT attenuated the inflammation in rats with chronic small intestinal mucositis

**FIGURE 2:**
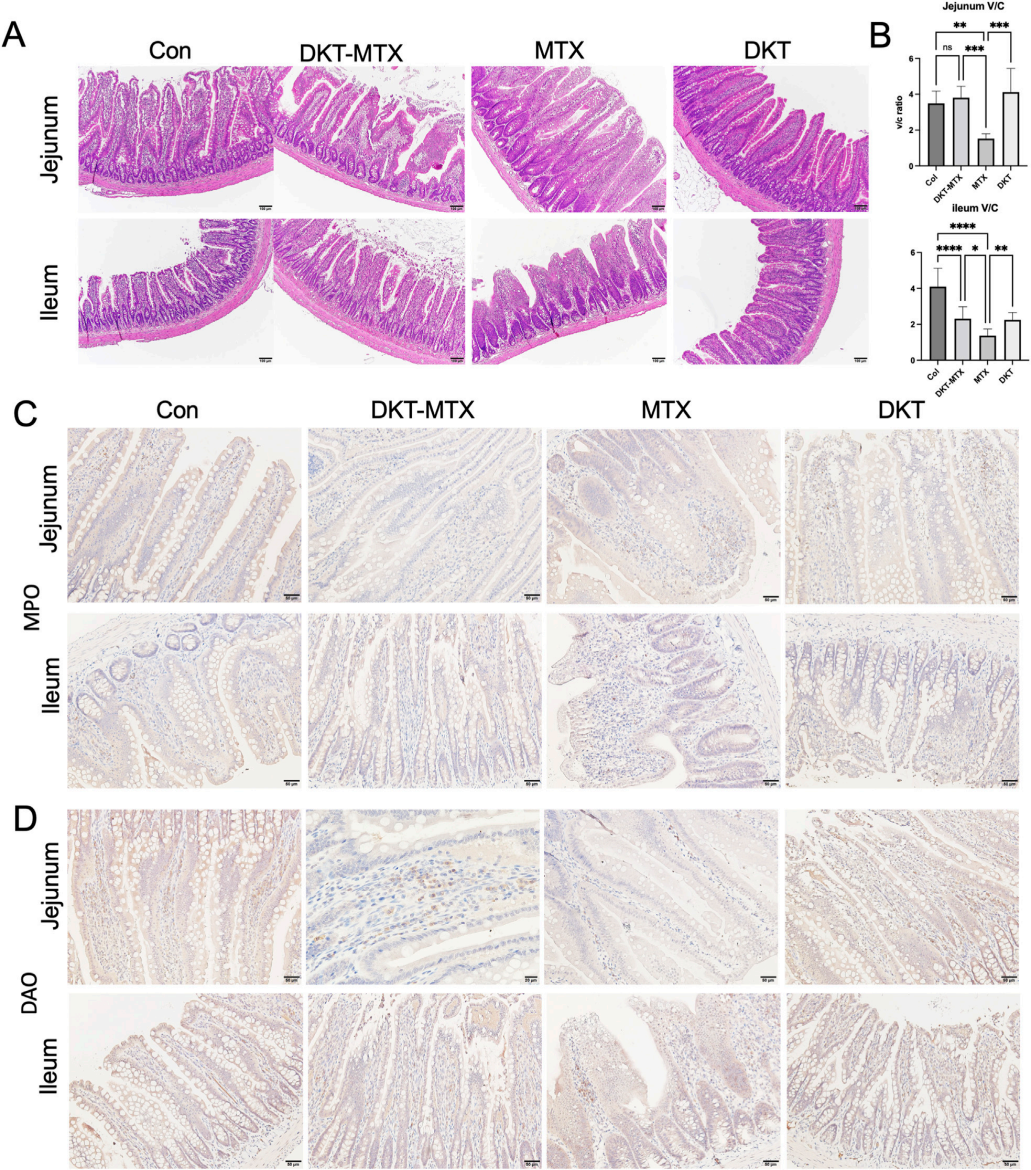
DKT attenuated inflammation in rats with chronic small intestinal mucositis. **(A)** HE staining showed the morphology of the intestinal mucosa. Scale bar = 100 μm. **(B)** DKT-MTX group had a higher ratio of villus to crypt length (V/C ratio) than the MTX group. The length was measure by Image J. (n > 10) Data represent the mean ± standard error of the mean. One-way ANOVA, *p < 0.05, **p < 0.01. ****p < 0.0001. IHC staining of MPO **(C)** and DAO **(D)**. The expression of MPO was downregulated in the DKT-MTX group and the expression of DAO was upregulated in the DKT-MTX group in comparison to the MTX group. Scale bar = 50 μm.

Pathological and inflammation-related factors were employed to evaluate inflammation of the small intestinal mucosa and the therapeutic efficacy of DKT in CIM. The HE staining results showed structural changes in the jejunum and ileum of the MTX group. These changes were characterized by villus atrophy, elongation of crypts, and infiltration of numerous eosinophilic granulocytes (Figure 2A). The ratio of villus height to crypt depth (v/c ratio) in the jejunum and ileum was reduced in the MTX group in comparison to the DKT-MTX group, with mean v/c ratio of 1.552 and 3.802, respectively (mean difference: 2.280; 95% CI: 0.8474 to 3.713,P Value = 0.0009) in the jejunum, and 1.372 and 2.31 in the ileum (mean difference: 0.9387; 95% CI: 0.1539 to 1.723, P Value = 0.0134) (Figure 2B). Myeloperoxidase (MPO) staining revealed activation of neutrophils and peroxidative damage in the small intestine, and immunohistochemistry indicated an increased number of positive cells on the mucosal surface of the small intestine in the MTX group in comparison to the DKT-MTX group (Figure 2C). Diamine oxidase (DAO), an intracellular enzyme in the villus layer of the small intestinal mucosa, plays a protective role by reflecting the maturity and integrity of the small intestinal mucosa. Immunohistochemistry indicated that the DKT-MTX group exhibited a higher number of DAO-positive cells on the mucosal surface of the small intestine in comparison to the MTX group (Figure 2D).

### 3.3 DKT promoted the repair of the mucosal barrier and nutrient absorption functions

**FIGURE 3:**
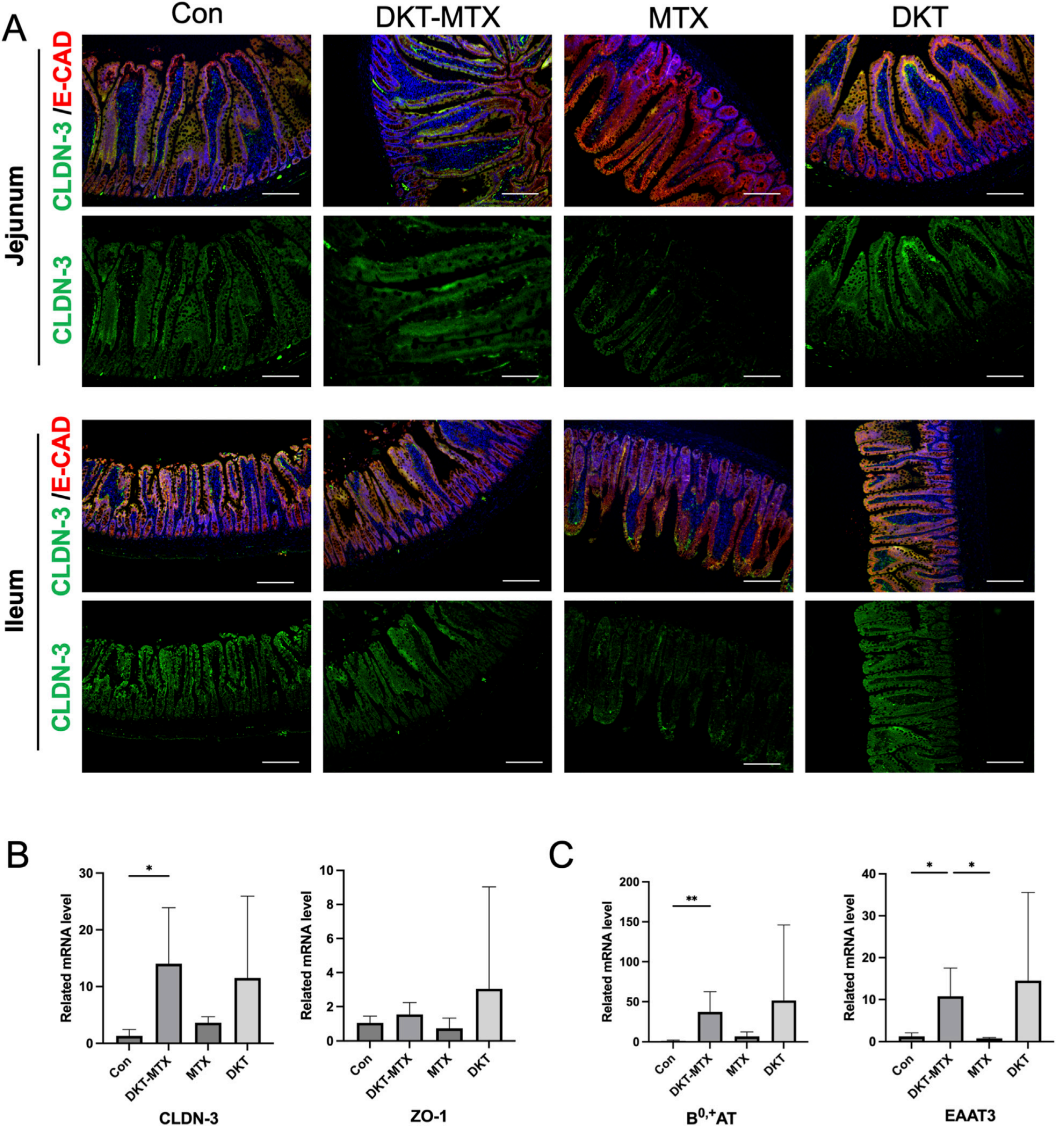
DKT promoted the repair of the mucosal barrier and nutrient absorption functions. **(A)** CLDN-3 and E-cad (mucosal barrier-associated tight junction proteins) were upregulated in the DKT-MTX group in comparison to the MTX group. Scale bar = 100 μm. At least three independent experiments were conducted, and the representative images were shown in this figure. **(B)** The QRT-PCR data showed that CLDN-3 and ZO-1 were upregulated in the DKT-MTX group. (n = 3–6) Data represent the mean ± standard error of the mean. One-way ANOVA, *p < 0.05, **p < 0.01. **(C)** BO-AT and EAAT3 (nutrient-related genes) were upregulated in the DKT-MTX group. (n = 3–6) Data represent the mean ± standard error of the mean. One-way ANOVA, *p < 0.05, **p < 0.01.

MTX induces mucosal barrier damage in the small intestine. Assessing the integrity of the small intestinal epithelium is instrumental for examining the protective effects of DKT against MTX-induced CIM. Immunofluorescence staining indicated that the expression of CLDN-3 in the jejunum and ileum was reduced in the MTX group in comparison to the DKT-MTX group, with no significant difference observed between the DKT and control groups (Figure 3A). The expression levels of E-CAD in the MTX and DKT-MTX groups did not differ to a statistically significant extent (Figure 3A). qRT-PCR of mRNA extracted from small intestinal mucosal tissues indicated the significant upregulation of the tight junction-associated gene CLDN-3 in the DKT-MTX group in comparison to the MTX group (Figure 3B). Additionally, there was no significant difference in the expression of ZO-1 (Figure 3B). The proteins B^0,+^AT, and EAAT3 are associated with epithelial and amino acid transport in the small intestinal mucosa and play a role in nutrient absorption. qRT-PCR revealed higher mRNA expression levels of B^0,+^AT, and EAAT3 in the small intestinal mucosa in the DKT-MTX group in comparison to the MTX group (Figure 3C).

**FIGURE 4:**
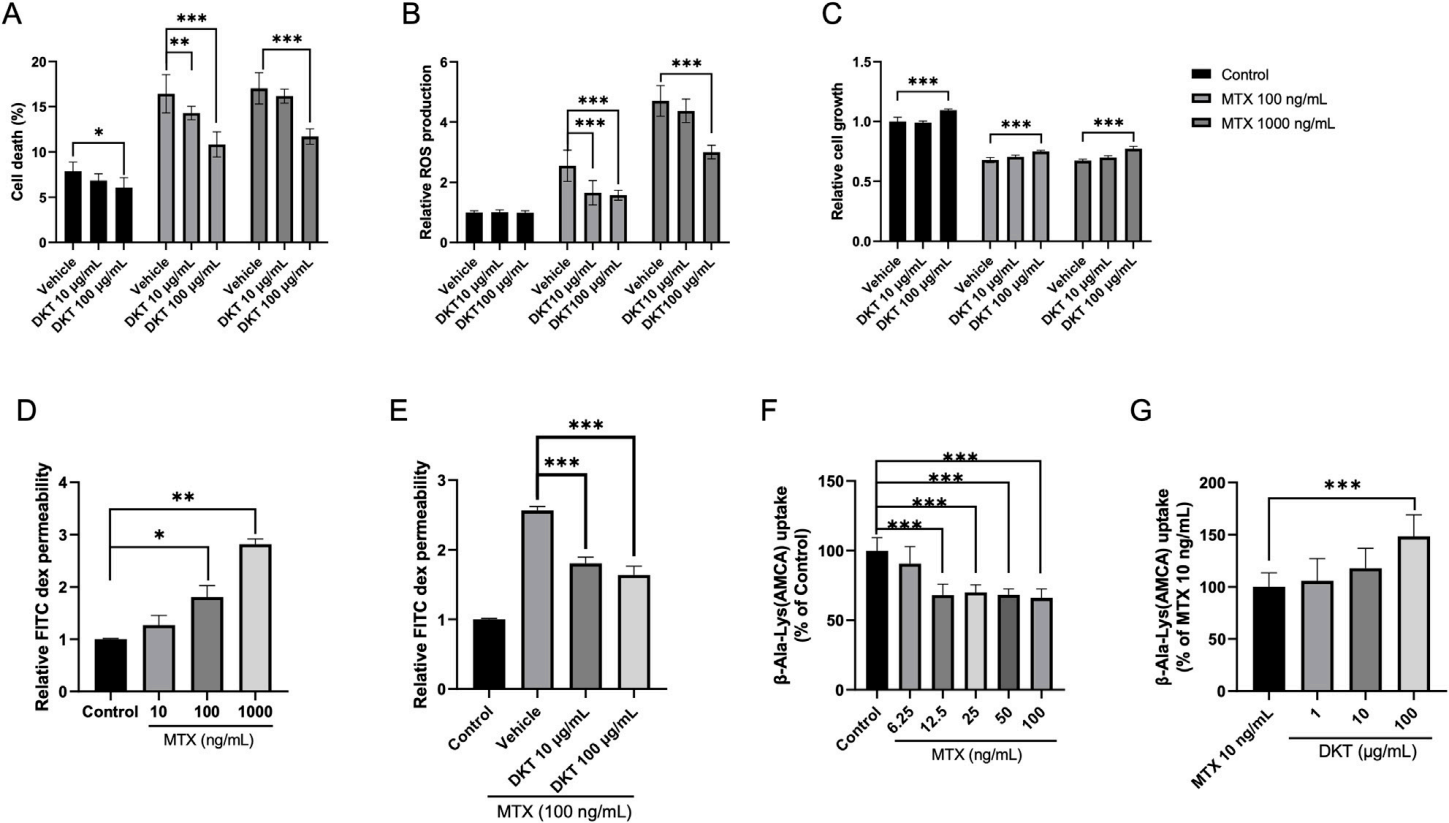
DKT protected intestinal epithelial cells from MTX, enhancing amino acid absorption and barrier function recovery *in vitro*. **(A)** Cell death percentage; MTX increase cell death, and it was suppressed by DKT co-treatment. **(B)** Relative ROS production; MTX increases ROS levels, and it was suppressed by DKT co-treatment. **(C)** Relative cell growth; MTX reduces growth and increased by DKT treatment. **(D)** Relative FITC dex permeability with increasing MTX doses. **(E)** Relative FITC dex permeability with DKT treatment at MTX 100 ng/mL. **(F)** β-Ala-Lys (AMCA) uptake decreases with increasing MTX. **(G)** β-Ala-Lys (AMCA) uptake with DKT treatment at MTX 10 ng/mL; uptake increases with higher DKT. Statistical significance is indicated with asterisks. Data are presented as mean ± SEM (n = 3). Statistical analysis was performed using Dunnett’s multiple comparisons test compared with the control group. *P < 0.05, **P < 0.01, ***P < 0.001.

To evaluate the direct effects of DKT on intestinal epithelial cells, *in vitro* experiments were performed with IEC-6 cells. MTX treatment significantly increased cell death and intracellular reactive oxygen species (ROS) levels, along with a marked suppression of cell proliferation. DKT treatment effectively attenuated MTX-induced cell death (Figure 4A) restored cell proliferation capacity (Figure 4C), and reduced ROS accumulation (Figure 4B). Furthermore, MTX treatment significantly reduced the barrier integrity of the monolayer in a dose-dependent manner (Figure 4D), and co-treatment with DKT effectively restored it (Figure 4E). We also examined amino acid uptake using β-Ala-Lys (AMCA) as a tracer *in vitro* using the intestinal epithelial cells of IEC-6 cells. MTX treatment led to a dose-dependent decrease in amino acid uptake (Figure 4F), while DKT co-treatment partially but significantly improved this uptake (Figure 4G).

### 3.4 DKT promoted the regeneration and repair of the small intestinal mucosa by enhancing angiogenesis

**FIGURE 5:**
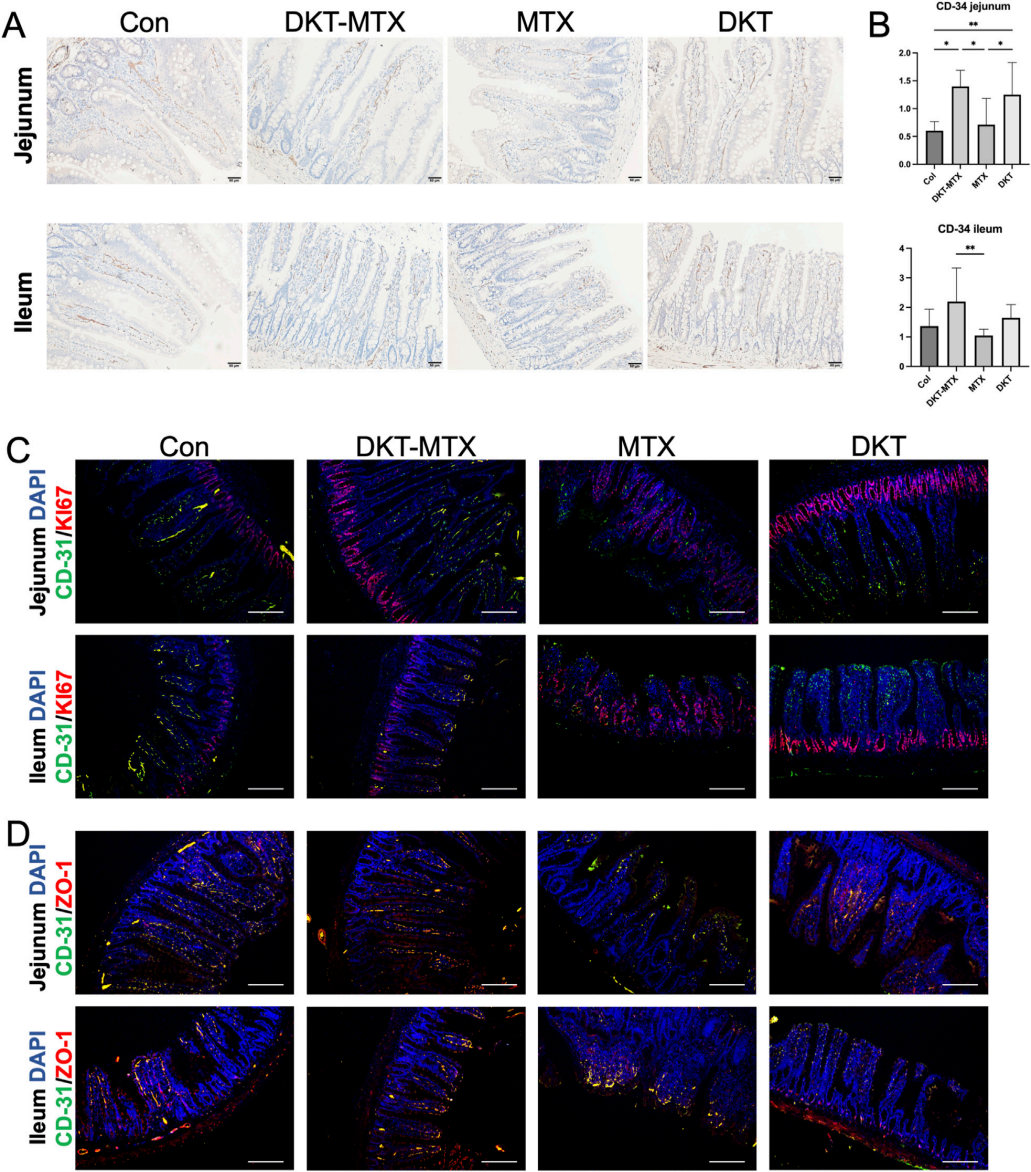
DKT promoted the generation of vasculature in the mucosa. **(A)** CD-34 immunohistochemical staining of blood vessels on the small intestinal mucosa. **(B)** The DKT-MTX and DKT groups had more CD-34-positive areas than the MTX group. **(C)** Immunofluorescence staining revealed that the expression levels of CD-31 and Ki-67 were higher in the DKT-MTX group than in the MTX group, and the location of Ki-67 in the MTX group was not limited to the crypts. Scale bar = 100 μm. **(D)** Results of immunofluorescence staining of CD-31 and ZO-1 in each group. Scale bar = 100 μm. At least three independent experiments were conducted, and the representative images were shown in this figure.

The regeneration and repair of the small intestinal mucosa depend on the blood supply within the mucosa. Immunohistochemical staining of CD34 demonstrated that the CD-34 positive area of the DKT-MTX group was larger than that of the MTX group, suggesting increased vascularity in the small intestine of the DKT-MTX group (Figures 5A,B). MTX-induced damage of the small intestine primarily involves inhibition of cell replication in the intestinal crypts, thereby disrupting the renewal of the intestinal mucosa, which typically occurs every 2–3 days and leads to mucosal injury. To explore the reparative effect of DKT on the small intestinal mucosal epithelium, immunofluorescence staining of Ki-67 showed a significant increase in Ki-67-positive cells in the DKT-MTX group in comparison to the MTX group, primarily concentrated within the crypts (Figure 5C). In contrast, in the MTX group, Ki-67-positive cells were not confined to the crypts, but were also present on the villi. The analysis of the results of immunofluorescence staining also demonstrated a positive correlation between Ki-67 and CD-31 (Figure 5C). Dual immunofluorescence staining of CD-31 and the mucosal barrier protein ZO-1 revealed a positive correlation (Figure 5D).

**FIGURE 6:**
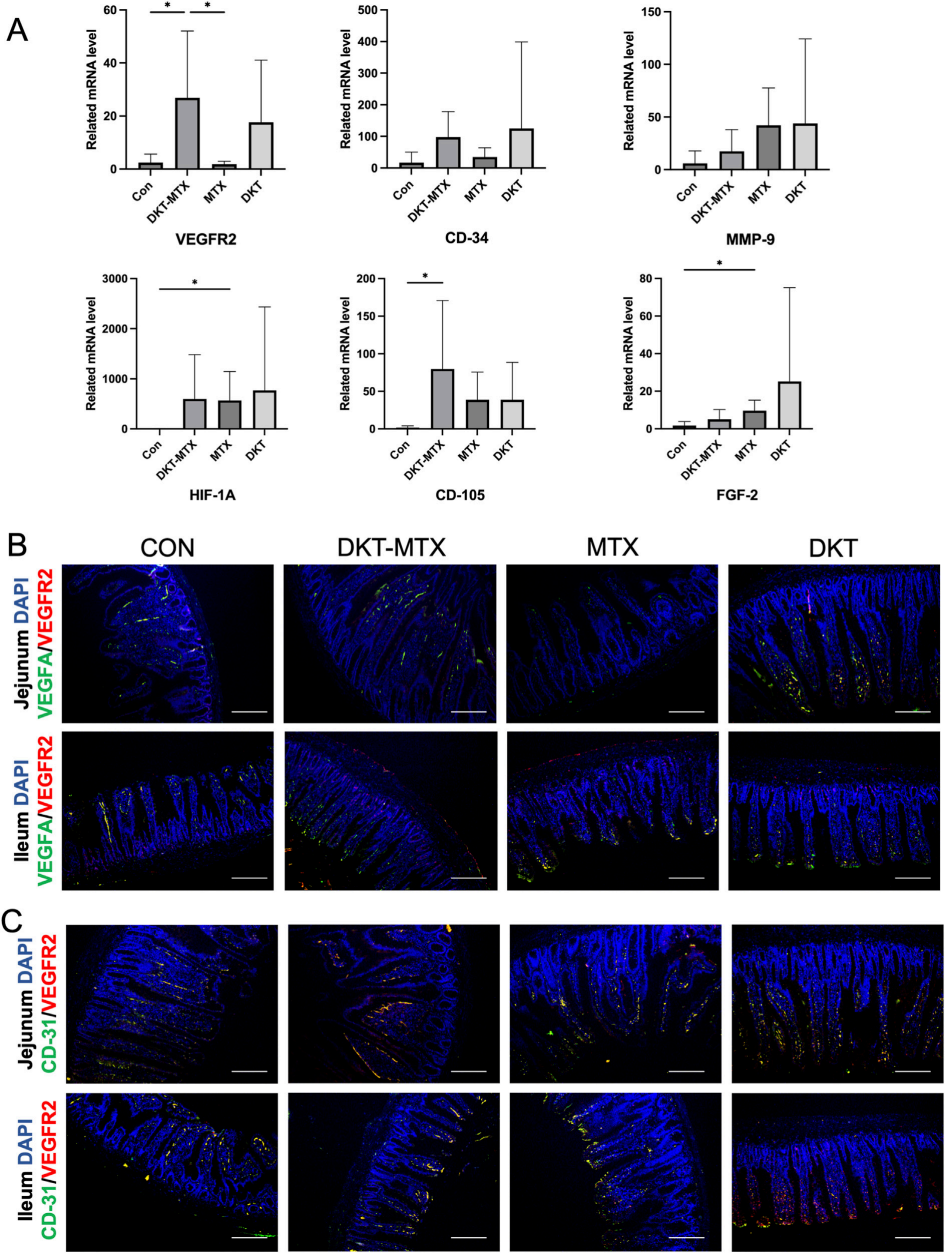
DKT promoted angiogenesis by upregulating angiogenic factor receptors. **(A)** QRT-PCR showed that angiogenesis-related genes were upregulated with by the administration of DKT. **(B)** Immunofluorescence staining of VEGFR2 and VEGFA in each group. Scale bar = 100 μm. **(C)** Immunofluorescence staining of VEGFR2 and CD-31 in each group. Scale bar = 100 μm. At least three independent experiments were conducted, and the representative images were shown in this figure.

To further investigate the specific mechanisms through which DKT promotes angiogenesis in the small intestinal mucosal epithelium, qRT-PCR of the intestinal tissue indicated the upregulation of genes related to angiogenesis in the DKT-MTX group in comparison to the MTX group (Figure 6A). Specifically, the expression levels of VEGFR-2, CD-34, and CD105 were elevated, with VEGFR-2 exhibiting a significant difference. Genes that were upregulated relative to the control group included HIF-1A, MMP-9, and FGF-2 (Figure 6A). qRT-PCR demonstrated that DKT upregulated the expression of VEGFR2, CD-34, CD105, HIF-1A MMP-9, and FGF-2, suggesting a role in promoting angiogenesis in the small intestinal mucosa (Figure 6A). Further immunofluorescence staining revealed that the DKT-MTX group exhibited higher expression levels of VEGFA and VEGFR-2 in comparison to the MTX group (Figure 6B). The dual immunostaining results demonstrated a correlation between CD31 and VEGFR-2, with higher expression levels observed in the DKT-MTX group than in the MTX group (Figure 6C).

## 4 Discussion

DKT, a drug with extensive therapeutic effects in the gastrointestinal tract, has been utilized in the treatment of various clinical conditions, including postoperative abdominal pain and abdominal fullness. In the context of drug-induced gastrointestinal mucosal injury, previous studies have demonstrated that DKT ameliorates MTX-induced acute injury to the small intestine (Li et al., 2023b). Furthermore, it has been shown to facilitate wound healing in the small intestine by upregulating VEGF (Wada et al., 2018). In this study, DKT exhibited protective effects against MTX-induced CIM. On one hand, it alleviated inflammation by reducing the neutrophil infiltration in the small intestinal mucosa, and on the other hand, it promoted the regeneration of the intestinal villi and repair of the mucosal barrier, synergistically upregulating amino acid transport-related genes B^0,+^AT and EAAT3 to enhance nutrient absorption. Mechanistically, DKT promoted vascular regeneration in the small intestinal mucosa by upregulating VEGFA and its mucosal receptors, VEGFR2, thereby facilitating the regeneration and repair of the mucosal epithelium. MTX-induced CIM primarily involves the inhibition of cell replication within its crypts, suppressing mucosal renewal and resulting in mucosal injury. A single dose of MTX typically induces mucosal damage in the small intestine for approximately 3 days, with the remaining 3 days serving as a repair cycle. Intermittent 6-day administration of MTX induces chronic damage to the small intestinal mucosa (Sukhotnik et al., 2018; Abolmaali et al., 2013). DKT mitigates inflammatory cell infiltration and reduces damage, primarily by enhancing mucosal repair by promoting vascular regeneration in the mucosa.

Critically ill patients and those receiving chemotherapy or radiation therapy show severe impairment of the intestinal barrier integrity (Chelakkot et al., 2018). One side effect of MTX, which is widely used in tumor chemotherapy and the treatment of rheumatic immune diseases, is damage of the small intestinal mucosa, which leads to mucosal barrier dysfunction (Song et al., 2006). In this study, the long-term use of low-dose MTX induced CIM in rats, leading to a decrease in CLDN-3 and ZO-1 proteins and damage to the barrier function of the small intestinal mucosa. The *in vitro* experiments of IEC-6 cell showed that DKT treatment could restore the barrier function induced by MTX, which related to the nutrient absorption. *In vivo*, MTX induces a decrease in the expression of amino acid transporters B^0,+^AT and EAAT-3 in the small intestinal mucosal epithelium, inducing damage to the mucosal nutrient absorption function. During the administration of MTX, DKT protects the structural and functional integrity of the small-intestinal mucosa. As for the main ingredient of DKT, the major active constituents of dried ginger (*Z. officinale* Roscoe) are gingerols and their dehydration products, shogaols. Among them, 6-shogaol is particularly abundant and pharmacologically potent, especially known for its anti-inflammatory and antioxidant effects (Wang et al., 2025). 6-Shogaol exhibits significant protective effects on the intestinal mucosa through multiple mechanisms (Jiao et al., 2023). It enhances epithelial barrier integrity by upregulating tight junction proteins (e.g., claudin-1, ZO-1, occludin) and reducing claudin-2 expression, suppresses inflammatory responses by inhibiting the NF-κB and TLR4 pathways, and mitigates oxidative stress via Nrf2 activation (Ouyang et al., 2022). Additionally, it reduces epithelial apoptosis through activation of the BDNF/TrkB/PI3K/AKT signaling pathway (Jiao et al., 2023; Li B. et al., 2023). These actions collectively help maintain mucosal integrity and reduce damage in models of colitis, fatty acid–induced injury, and ischemia-reperfusion. Ginsenosides Rb1, Rh4, and Re can restore intestinal barrier dysfunction by regulating the gut microbiota (Yang et al., 2021; Bai et al., 2021; Zhang et al., 2018). In addition, ginsenoside Rk3 can enhance the expression of tight junction proteins and inhibit the expression of inflammatory cytokine proteins (TNF-α, IL-1β, and IL-6) to promote recovery of the barrier function (Chen et al., 2021). Total ginsenosides promote IEC-6 cell proliferation by affecting the regulatory mechanism mediated by polyamines, which contribute to the recovery of the barrier function after injury (Zhu et al., 2021). Other studies have reported similar effects of DKT in promoting mucosal barrier repair (Takasu et al., 2017). The integrity of the small intestinal mucosa is the structural basis for nutrient absorption, and the transporters B^0,+^AT, (SLC7A9) form a complex with rBAT (related to b0,+ amino acid transporter, SLC3A1) in the apical membrane and mediate the import of cationic amino acids and cystine in exchange for neutral amino acids (Bröer, 2023). EAAT3 (SLC1A1) mediates the active uptake of anionic amino acids through a complex transport mechanism involving Na+ and K+ ions (Bröer, 2023). The expression of B^0,+^AT and EAAT3 was significantly increased in the DKT-MTX group in comparison to the MTX group, which also supports that DKT could restore the small intestinal mucosal function. The results of this study also concluded that DKT can protect and promote the repair of the mucosal barrier in MTX-induced chronic inflammation of the small intestinal mucosa.

Normal small intestinal mucosal functions, including nutrient absorption, the immune barrier function, and secretory functions, require the support of a normal and healthy vasculature. Manieri et al. suggested that a poor angiogenic response after mucosal injury is a common mechanism leading to penetrating ulcers in mice and humans (Manieri et al., 2015). The promotion of angiogenesis can stimulate intestinal healing (Manieri et al., 2015). Previous studies reported that DKT can increase the expression of VEGFA in small intestinal tissue and promote anastomotic healing (Parvadia et al., 2007). Meanwhile, in DKT, the ingredient 6-shogaol (6SG), has been shown to upregulate the blood flow in the rat small intestine by increasing epithelial transient receptor potential ankyrin 1 (TRPA1)-dependent adrenomedullin release in a dose-dependent manner (Kono et al., 2013). The restoration of blood flow also promotes the repair of ulcerative lesions in the gastrointestinal tract (Sørbye and Svanes, 1994). In this study, DKT promoted the repair of MTX-induced CIM in the small intestinal mucosal epithelium, in which Ki-67-positive cells in the small intestinal mucosal barrier and crypts were positively correlated with the blood vessel density in tissue. Further experiments demonstrated that DKT upregulated the gene expression of VEGFR2 and VEGFA content in tissues. FGF2 and CD-105, which are involved in angiogenesis, were upregulated by DKT. The dense capillary network is maintained through continuous VEGFA signaling by activating the receptors VEGFR-1/VEGFR-2, which also promotes endothelial cell fenestration, ensuring efficient nutrient uptake (Shibuya, 2011). In this experiment, DKT promoted an increase in the expression of VEFGR-2 and VEGF-A, and immunofluorescence staining of VEGF-A and VEGFR-2 showed partial co-localization, which plays a certain role in promoting vascular proliferation. Repaired mucosal epithelial cells may further promote and polarize vascular growth within the villi (Shibuya, 2011). Villus blood capillaries are VEGFA-dependent and display signs of angiogenesis. Studies have shown that in addition to promoting angiogenesis, VEGF and its homologues can also promote the division of Paneth cells in the small intestine and promote the division, differentiation, and migration of stem and progenitor cells in the small intestine (Schlieve et al., 2016). Therefore, DKT could promote the repair of the intestinal mucosa and villi in MTX-induced CIM by upregulating the expression of VEGFA and VEGFR, as well as other factors related to angiogenesis.

It appears that DKT did not significantly improve the body weight of the rats. This was different from our previous experience with acute and severe small intestinal mucosal injury induced by MTX in rats (Wada et al., 2022). However, it has demonstrated notable therapeutic effects in other aspects, including reducing mucosal damage, decreasing mucosal inflammation, promoting mucosal repair, and improving symptoms. Moreover, it effectively ameliorated long-term chronic mucosal damage induced by MTX, thereby suggesting a potential role in reducing MTX-related mortality. The improvement in rat mucosal damage may be attributed to a reduction in fluid loss, as evident in mucous stools. This may be due to DKT enhancing the mucosal barrier in the small intestine, thereby reducing mucous exudation. The extent of neutrophil infiltration, represented by MPO in the tissue, was reduced by DKT. This indicates that DKT mitigated the neutrophil infiltration that was induced by MTX. DAO, a highly active intracellular enzyme in the villi of the human and mammalian small intestine, especially at the tip of the villi, plays a role in the metabolism of histamine and various polyamines (Fukudome et al., 2014). Immunohistochemistry demonstrated a significant elevation in DAO levels in the DKT-MTX group compared to those in the MTX group. This outcome suggests that the small intestinal villi in the DKT-MTX group were relatively more mature with higher metabolic activity. This was also demonstrated by the higher villus-to-crypt ratio in the small intestinal mucosa of the rats in the DKT-MTX group. The recent studies suggested that botanical drugs hold great potential as adjuvant therapy for the prevention and treatment of chemotherapy-induced side effects, especially the gastroenterological side effects which could lead to the chemotherapy regimen adjustment or discontinuation (Okem et al., 2023; Ohnishi and Takeda, 2015). While intestinal protection is critical for treatment continuity, preserving antineoplastic efficacy remains paramount. While DKT ameliorates MTX-related gastrointestinal toxicity, it is effective in maintaining the efficacy of MTX in chemotherapy or the treatment of rheumatoid arthritis, thereby reducing the dose reduction or discontinuation of the drug due to its side effects.

This study has several limitations. First, the assessment of diarrhea severity was based on subjective observation rather than quantitative scoring, which may introduce observer bias. Second, only a single dose of DKT, based on its clinically used concentration, was tested in this study, and the lack of a dose-response analysis limits our understanding of its optimal therapeutic range. Although *in vitro* experiments used DKT concentrations ranging from 10 to 100 μg/mL, the correlation between these concentrations and the *in vivo* dosage remains unclear. Third, although the study demonstrated beneficial effects of DKT, the specific active metabolites responsible for these effects were not identified due to the complex nature of the botanical formulation. Also, DKT (Lot ID:2180100010, 2220100010) used in this study was prepared in accordance with the standards of the Japanese Pharmacopoeia; however, batch-specific component profiles analyzed by methods such as HPLC or LC-MS have not been obtained. Therefore, the detailed component profile of the specific batch used in this study remains unknown. Finally, long-term safety, pharmacokinetics, and potential drug interactions of DKT under chronic administration were not investigated and should be explored in future studies. These limitations highlight several important areas that should be addressed in future research. Future studies should aim to further elucidate the specific active components of DKT responsible for its therapeutic effects, potentially through fractionation or purified compound testing. Dose–response studies are also needed to determine the optimal therapeutic window and assess potential toxicity at higher concentrations. In addition, mechanistic investigations using genetic or pharmacologic inhibition of angiogenic pathways could provide deeper insights into how DKT promotes mucosal repair. Finally, clinical trials will be essential to validate the translational potential of DKT in managing methotrexate-induced intestinal mucositis and other gastrointestinal complications.

In conclusion, DKT may exert a therapeutic effect against MTX-induced CIM by stimulating cell proliferation, promoting vascular regeneration through the upregulation of angiogenesis-related factors, and enhancing mucosal regeneration along with restoration of the mucosal barrier function. Despite containing many bioactive metabolites and having multi-target effects, DKT has been shown to applicable in the prevention and treatment of intestinal injuries.

## Data Availability

The raw data supporting the conclusions of this article will be made available by the authors, without undue reservation.
