# Supplementary figures and images for: Daikenchuto improves methotrexate-induced chronic small intestinal mucositis by promoting angiogenesis
Source: Front Pharmacol. 2025 Aug 21;16:1623726. doi: 10.3389/fphar.2025.1623726 (PMC12409372; doi:10.3389/fphar.2025.1623726)

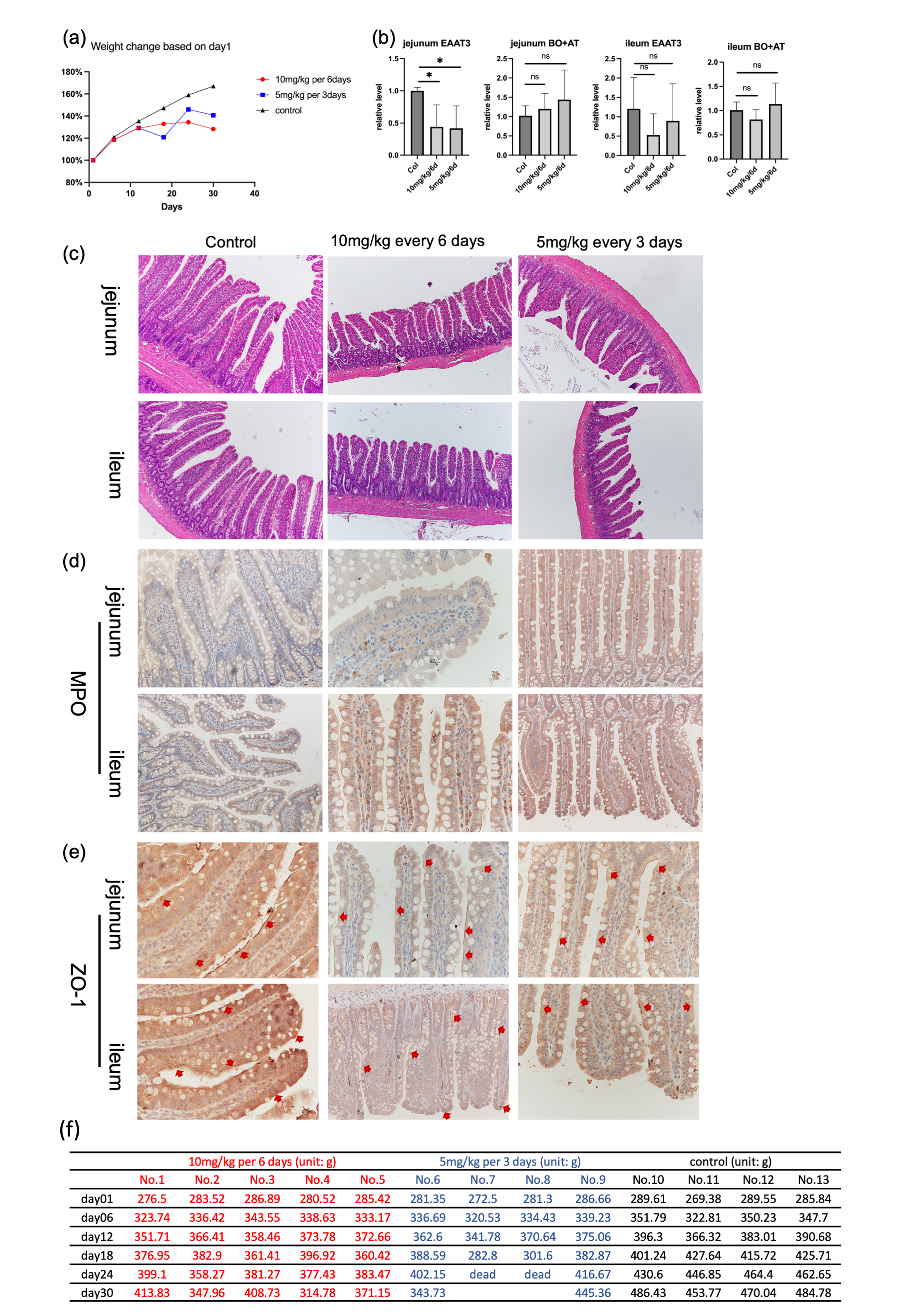

Supplement: Supplementary file 1 [file Image1.tiff]

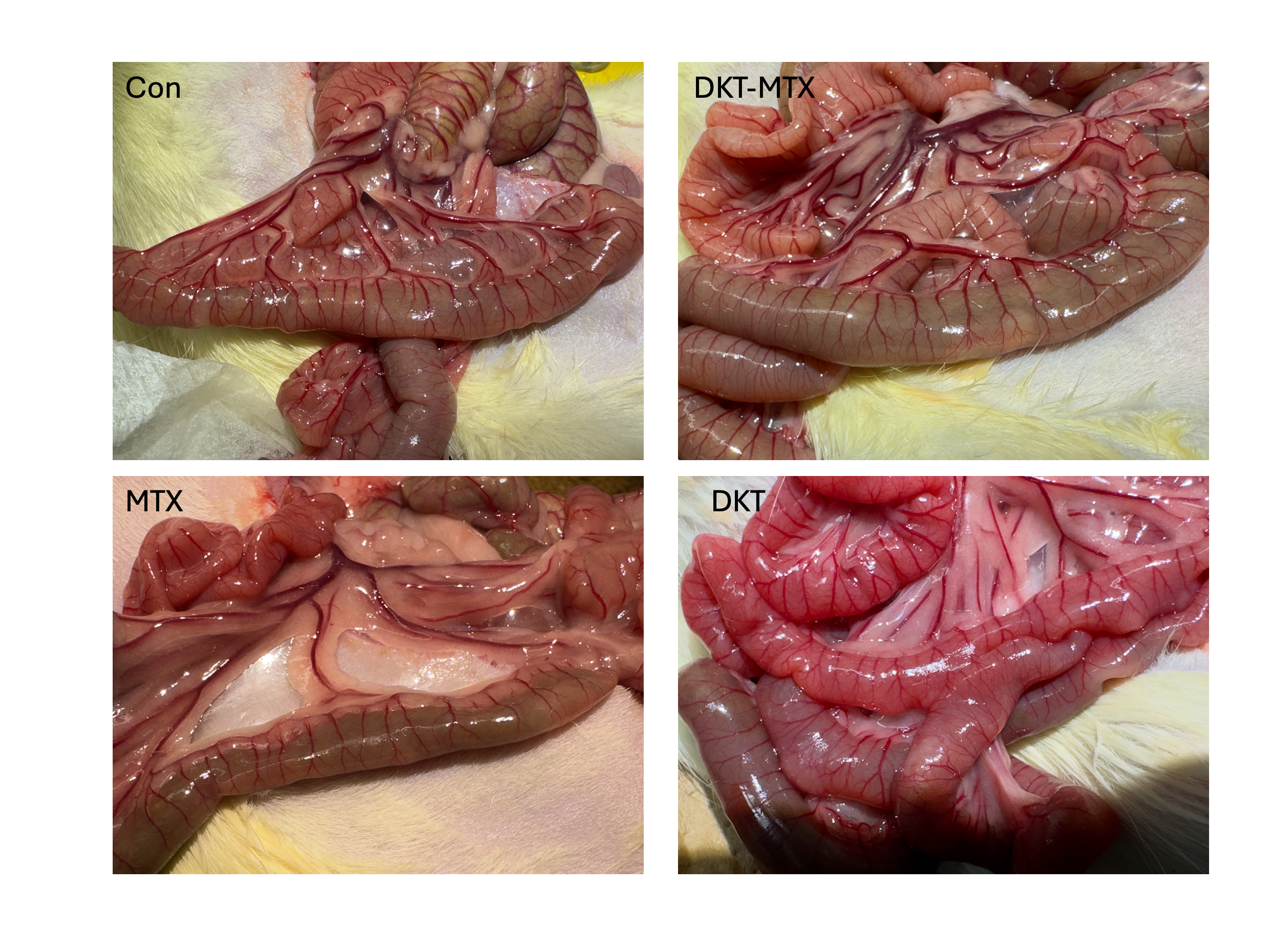

Supplement: Supplementary file 3 [file Image2.tiff]
