# Supplementary material for: Daikenchuto improves methotrexate-induced chronic small intestinal mucositis by promoting angiogenesis
Source: Front Pharmacol. 2025 Aug 21;16:1623726. doi: 10.3389/fphar.2025.1623726 (PMC12409372; doi:10.3389/fphar.2025.1623726)
Supplement: Supplementary file 2 [file Table1.docx]

Table s1. List of the first and secondary antibodies in the experiments.

| MPO antibody | ab9535 | Abcam |
| --- | --- | --- |
| Rat diamine oxidase (DAO) antibody | MBS2025695 | MYBiosource,Inc |
| CD34 | ab81289 | Abcam |
| CD31 | ab281583 | Abcam |
| VEGFR-2 | ab2349 | Abcam |
| VEGFR-3 | ab27278 | Abcam |
| KI-67 | ab16667 | Abcam |
| ZO-1 | ab221547 | Abcam |
| CLDN-3 | ab231303 | Abcam |
| VEGFA | ab52917 | Abcam |
| Anti-rabbit IgG-peroxidase | A0545 | Sigma-Aldrich Co. LLC. |
| Anti-mouse IgG-peroxidase | A9044 | Sigma-Aldrich Co. LLC. |
| Anti-rabbit IgG-TRITC | T6778 | Sigma-Aldrich Co. LLC. |
| Anti-Mouse IgG-FITC | F9137 | Sigma-Aldrich Co. LLC. |
| Anti-Mouse IgG-TRITC | T2402 | Sigma-Aldrich Co. LLC. |
| Anti-rabbit IgG-FITC | F9887 | Sigma-Aldrich Co. LLC. |

| Gene Aliases | Gene symbol | Gene name | TaqMan® ID | Amplicon Length | Target species |
| --- | --- | --- | --- | --- | --- |
| Gapdh | Gapdh | Glyceraldehyde-3-phosphate-dehydrogenase | Rn99999916_s1 | 87 | Rat |
| B^0,+^AT | Slc7a9 | Solute carrier family 7 (Amino Acid Transporter Light Chain, Bo, + System) | Rn00588400_m1 | 73 | Rat |
| EAAT3 | Slc1a1 | Broad neutral amino acid transporter | Rn00564705_m1 | 92 | Rat |
| ZO-1 | Tjp1 | Tight junction protein 1 | Rn07315717_m1 | 64 | Rat |
| VEGFR-2 | Kdr | Kinase insert domain receptor | Rn00564986_m1 | 75 | Rat |
| CD-34 | CD34 | CD34 molecule | Rn03416140_m1 | 60 | Rat |
| CLDN-3 | CLDN-3 | Claudin 3 | Rn00581751_S1 | 90 | Rat |
| MMP-9 | MMP9 | Matrix metallopeptidase 9 | Rn00579162_m1 | 72 | Rat |
| HIF-1A | HIF-1A | Hypoxia-inducible factor 1 | Rn01472831_m1 | 90 | Rat |
| CD-105 | ENG | Endoglin | Rn01438763_m1 | 81 | Rat |
| FGF-2 | FGF2 | Fibroblast growth factor 2 | Rn00570809_m1 | 63 | Rat |

Table s2: list of the Taq-man primer of Rt-PCR performed in the experiments.
